# One in a Million: Genetic Diversity and Conservation of the Reference *Crassostrea angulata* Population in Europe from the Sado Estuary (Portugal)

**DOI:** 10.3390/life11111173

**Published:** 2021-11-03

**Authors:** Stefania Chiesa, Livia Lucentini, Paula Chainho, Federico Plazzi, Maria Manuel Angélico, Francisco Ruano, Rosa Freitas, José Lino Costa

**Affiliations:** 1 Department of Molecular Sciences and Nanosystems, Ca’ Foscari University of Venice, 30172 Venice, Italy; 2 ISPRA—The Italian Institute for Environmental Protection and Research, 00144 Rome, Italy; 3 Department of Chemistry, Biology and Biotechnologies, University of Perugia, 06123 Perugia, Italy; livia.lucentini@unipg.it; 4 MARE—Marine and Environmental Sciences Centre, Faculdade de Ciências da Universidade de Lisboa, 1749-016 Lisboa, Portugal; pmchainho@fc.ul.pt (P.C.); jlcosta@fc.ul.pt (J.L.C.); 5 ESTSetubal-CINEA, Instituto Politécnico de Setúbal, Estefanilha, 2910-761 Setúbal, Portugal; 6 Department of Biological, Geological and Environmental Sciences, University of Bologna, 40126 Bologna, Italy; federico.plazzi@unibo.it; 7 Department of Sea and Marine Resources, IPMA—Portuguese Institute of Sea and Atmosphere, 1495-006 Lisboa, Portugal; mmangelico@ipma.pt (M.M.A.); fruano@ipma.pt (F.R.); 8 Department of Biology and CESAM, University of Aveiro, 3810-193 Aveiro, Portugal; rosafreitas@ua.pt

**Keywords:** *Crassostrea angulata*, Portuguese oyster, mtDNA, *cox1*, phylogeography, phylogenetics, haplotype diversity, oyster conservation, genetic diversity

## Abstract

The production of cupped oysters is an important component of European aquaculture. Most of the production relies on the cultivation of the Pacific oyster *Crassostrea gigas*, although the Portuguese oyster *Crassostrea angulata* represents a valuable product with both cultural and economic relevance, especially in Portugal. The authors of the present study investigated the genetic diversity of Portuguese oyster populations of the Sado estuary, both from natural oyster beds and aquaculture facilities, through *cox1* gene fragment sequencing. Then, a comparison with a wide dataset of cupped oyster sequences obtained from GenBank (up to now the widest available dataset in literature for the Portuguese oyster) was performed. Genetic data obtained from this work confirmed that the Pacific oyster does not occur in the natural oyster beds of the Sado estuary but showed that the species occasionally occurs in the oyster hatcheries. Moreover, the results showed that despite the founder effect and the bottleneck events that the Sado populations have experienced, they still exhibit high haplotype diversity. Risks are arising for the conservation of the Portuguese oyster reference populations of the Sado estuary due to the occurrence of the Pacific oyster in the local hatcheries. Therefore, researchers, local authorities, and oyster producers should work together to avoid the loss of this valuable resource.

## 1. Introduction

Oyster farming has a high economic relevance in the European economy, mainly relying on cupped oyster production. The European production of cupped oysters was 93,103 tons in 2014, approximately 2% of the worlds production, with France, Ireland, and the Netherlands being the main producers (96%) [1]. EU production reached 108,910 tons in 2008 before severe outbreaks of pathogens, including a *Herpes* virus, that struck French production and spread to all shellfish-producing European countries including Portugal. In 2015, production started to rise again, reaching 110,000 tones the following year [1]. The largest production increases have been observed in Ireland and Portugal, who target the French market. Half of the spat used for oyster farming is supplied by hatcheries; the remaining 50% is wild spat collected by farmers.

Concerning their taxonomy, cupped oysters belong to two identified sister species [2]: the Pacific oyster *Crassostrea gigas* (Thunberg, 1793) and the Portuguese oyster *Crassostrea angulata* (Lamarck 1819), both introduced in Europe from their native ranges in the Northwestern Pacific [2].

The taxonomic classification of cupped oysters has been debated for almost two decades, being identified as a single species or two depending on their cross-fertilization and the genetic variation estimated by different molecular markers—for a complete list of references, see [2]. Recent genomic studies [2,3] have reinforced the hypothesis of two genetically similar but differentiated species. Previous studies [2] have stated that the two species exhibit partial reproductive isolation but also genetic introgression as a result of secondary contacts in the areas where both species have been introduced.

Nowadays, the consensus is that the Portuguese oyster was the first to be introduced in Europe. It was accidentally introduced by Portuguese merchants during the 16th century, probably from Taiwan [2,4,5]; however, it is impossible to establish where the original stocks came from [2,6]. Following the first accidental introduction in Portugal, by the end of the 19th century, the species was already occurring in France, where it was voluntarily introduced for farming and exploitation—see [2] and references therein. Moreover, the Portuguese oyster was introduced in other European countries for shellfish farming, replacing the native flat oyster *Ostrea edulis* (Linnaeus, 1758) (see [2] and references therein). *C. angulata* had a high economic relevance in European aquaculture until the late 1970s, when it practically disappeared due to high mortality rates [7].

The main cause of such a massive mortality, which almost led to the extinction of the species, was associated with the rapid and severe degradation of the main oyster bed ecosystems. In Portuguese systems such as the Sado and Tagus estuaries, this was caused by an impressive development in industrial fabric on the Lisbon and Setúbal water fronts. A second cause was related to the occurrence of a severe pathology, a gill disease characterized by a severe lesion frame in the gills and mantle tissues. The disease was first described by Alderman in 1969 [8] and later associated with the presence of a pathogen identified by Comps in 1976 as an iridovirus [9].

The consequence of such events was the decline of an important oyster industry that permanently employed more than 5000 people, especially in the Sado and Tagus estuaries. The Pacific oyster was then introduced to replace Portuguese oyster cultivation, and it is currently the main species supporting oyster production in Europe (for details on historical and oyster production data in Portugal, see [10,11]). A few populations of *C. angulata*, mainly located in Southern Europe and Northern Africa, survived the massive mortality events of the 1970s and the introduction of *C. gigas* [12]; these populations nowadays occur in Portugal, Spain, and Morocco [2,13,14].

It is worth mentioning that very recent genomic data [2] highlight that the Portuguese population of the Sado estuary represents the reference *C. angulata* population in Europe due to its low level of genetic introgression with the Pacific oyster. Therefore, special attention should be paid to the management and conservation of this valuable Portuguese oyster population. Even if *C. angulata* cannot be strictly considered to be a native species, it has a relevant commercial and cultural value in Portugal, where it has been exploited for over a century and is now considered a valuable natural resource.

Despite this evidence, there has been a lot of pressure put on aquaculture producers to increase the production of the Pacific oyster in both the Sado and Mira estuaries, similarly to what happened in other areas of the Portuguese coast, such as the Ria Formosa and Ria de Aveiro lagoons [15]. The genetic introgression of the Portuguese oyster with Pacific oyster has already been confirmed in Ria Formosa [2], posing risks for the conservation of *C. angulata* wild populations.

Therefore, the specific genetic characterization of both natural and farmed cupped oyster populations of the Sado estuary was performed in this study, with the aim of contributing to better management and conservation plans for this valuable resource in Portugal and Europe in general.

## 2. Materials and Methods

### 2.1. Study Area

The Sado estuary is located in Southern Portugal, covering a total area of 180 km^2^, with a mean river flow of 40 m^3^·s^−1^ and a mean depth of 6 m [14,16]. The estuary hosts both a shipping port and recreational marinas 14], but it also represents one of the most important sites for aquaculture production, especially of oysters, in the country. Wetlands, intertidal mudflats, and saltmarshes are predominant habitats [14] (and references therein), mostly characterized by sandy bottoms [14,17]. Sandy and muddy bottom habitats have high invertebrate species richness, and NIS (non-indigenous species) have also been detected [14].

### 2.2. Oyster Collection

**Figure 1:**
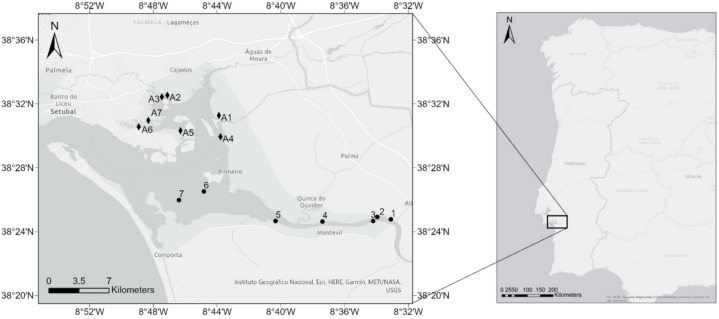
Sampling locations of *C. angulata* from seven natural oyster beds (black dots) and seven aquaculture facilities (black diamonds) in the Sado estuary, Portugal.

Oyster collection was carried out in 2015 from Portuguese natural oyster beds and aquaculture facilities. The collection from natural oyster beds was conducted at seven sites along the estuary salinity gradient, whilst the collection of farmed samples was conducted in seven aquaculture facilities located in the estuarine region (Figure 1).

For each sampling site, whether from natural oyster beds or aquaculture, 20 individuals were collected. The adductor muscle was dissected in each specimen, individually fixed in absolute ethanol, and preserved at −20 °C until DNA extraction and purification. Ten additional samples of *C. gigas* were also collected in the Ria de Aveiro lagoon as reference material (40°69′ N, 8°69′ W).

### 2.3. HMW DNA Extraction and Purification

High molecular weight (HMW) total genomic DNA was extracted and purified for each sample from the adductor muscle fixed in absolute ethanol with the DNeasy Blood & Tissue Kit (Qiagen, Hilden, Germany) following the manufacturer’s instructions, and its quality and quantity were verified via an electrophoretic run in 1% agarose gel and TAE buffer (1×).

### 2.4. Cox1 Gene Fragment Amplification and Sequencing

A fragment of the mitochondrial cytochrome coxidase subunit I (*cox1*) gene was amplified by PCR using the universal primers *LCO1490* (5′-GGTCAACAAATCATAAAGATATTGG-3′) and *HCO2198* (5′-TAAACTTCAGGGTGACCAAAAAATCA-3′) [18] and the specific PCR conditions developed for oysters [19].

The amplification reactions were performed in a total volume of 25 µL, including 20.375 µL of sterilized distilled water, 2.5 µL of a 5× colorless reaction buffer, 0.75 µL of MgCl_2_ (50 mM), 0.25 µL of each primer (10 pmol/µL), 0.5 µL of dNTP mixture (10 mM), 0.125 µL of Taq polymerase (Enzytech, Roche Diagnostics, Mannheim, Germany), and 0.25 µL of DNA.

PCR was carried out for 4 min at a denaturation temperature of 95 °C, followed by 40 cycles of 1 min at 95 °C, 1 min at 45 °C, 2.5 min at 72 °C, and a final extension of 7 min at 72 °C.

The quality of PCR products was verified with an electrophoretic run on a 2.5% agarose gel and 1× TAE buffer, and they were visualized under UV light: amplification products exhibited a molecular weight of about 650 bp. The PCR products were then purified by Promega Wizard™ SV Gel and PCR Clean-Up System (Promega, Madison, WI USA), following the standard protocol; finally, Sanger sequencing was conducted by STAB Vida, Caparica, Portugal.

### 2.5. Phylogenetic Analyses and Haplotype Analysis

Electropherograms were visualized in Mega X (https://www.megasoftware.net/, accessed on 2 January 2021) and imported into a multiple sequence alignment [20]. The sequences obtained were compared with all *C. angulata* available in GenBank up to December 2020, and those of other species of the genus *Crassostrea*—*C. gigas*, *C. dianbaiensis*, *C. sikamea*, *C. nippona*, *C. virginica*, *C. ariakensis*—available on GenBank (Table S1); moreover, *Saccostrea glomerata* and *S. cucullata* were used as outgroups in the final alignment (Table S1).

Sequences were aligned using amino acids as a guide through the TranslatorX server [21] using the Muscle [22,23] algorithm and the invertebrate mitochondrial genetic code, with no alignment cleaning. Sites with low or noisy phylogenetic signal were masked using Gblocks 0.91b [24]: the minimum number of sequences for a flank position was set to 50% + 1, the maximum number of contiguous no conserved positions was set to 10, the minimum length of a block was set to 5, and all gap positions were allowed. The aligned *cox1* fragment was split into the three codon positions thanks to a custom-tailored Python script (available from FP upon request), which resulted in three datasets: cox1_1, cox1_2, and cox1_3; these datasets were concatenated into the final alignment. A phylogenetic tree was inferred using IQ-TREE 1.7-beta7 [25] with 1000 ultrafast bootstrap replicates [26]. ModelFinder [27] was used to select substitution models; the greedy strategy was chosen to select the best partitioning scheme [28,29].

In order to estimate the degree of saturation in our dataset, the substitution saturation test developed by Xia and colleagues [30,31] was applied. Eventually, the EMBOSS 6.6.0 distmat application [32] was used to compute pairwise (uncorrected) p-distances to be plotted over pairwise ML distances computed in RAxML 8.2.12 [33].

The PopART v 1.7 software [34] was used to draw the minimum spanning network by selecting the statistical parsimony criterion and setting ε = 0. The sequences were also analyzed using statistical parsimony performed in [35] tested through the TCS v.1.21 program [36], in which we set the network connection limit at 90% and gaps as “missing”. TCS allowed for the identification of different haplotypes, desegregating them in haplogroups. TCS produced networks that clarified the relationships between different haplotypes/haplogroups, showing the significant number of substitutions connecting haplotypes. The network was visualized and plotted with tcsBU [37]. Spatial or demographic expansion was estimated through the Tajima D neutrality test [38] using the DNAsp 5.0 program [39]. Tajima’s D statistic tested the departure from neutrality by measuring the differences between the average number of pairwise nucleotide differences and the number of segregating sites [38]. If both balancing or purifying selection were absent, only the population expansion significantly lowered Tajima’s D to zero; the positive increase of this statistics may be related to a population bottleneck [38].

## 3. Results

The final alignment included 394 *cox1* sequences, 110 of which were the original sequences collected in this study in the Sado estuary (Table S2) from seven natural oyster beds and seven aquaculture facilities. The total alignment length was 543 base pairs (bps).

### 3.1. Phylogenetic Analysis

No significant saturation was detected across the three codon positions; therefore, they were all retained for subsequent analyses (Figure S1 and Table S3).

**Figure 2:**
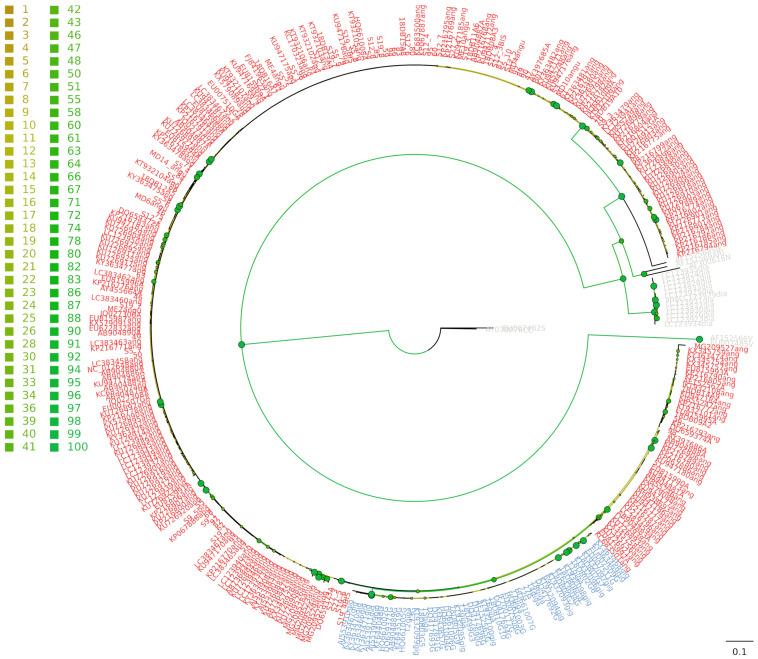
Maximum likelihood phylogenetic tree. Red, *C. angulata*; blue, *C. gigas*; grey, outgroup. The color of the dots depicted at nodes indicates ultrafast bootstrap values (light brown = 0%; solid green = 100%). Correspondence between bootstrap values and colors are reported as a legend.

The *C. angulata* and *C. gigas* species resulted in a single monophyletic clade, with ultrafast bootstrap support (UFboot) equal to 100 (Figure 2). Moreover, *C. gigas* was recovered as monophyletic (UFboot = 100) within a wide polytomy of *C. angulata* OTUs, where the phylogenetic relationships were not completely resolved (Figure 2). The *C. gigas* clade was retrieved as the sister group of the *C. angulata* clade (UFboot = 82), which entirely comprised Pacific specimens, but the statistical support of the node was low (UFboot = 72).

All the original samples analyzed within this study from the Sado estuary were nested within *C. angulata*, except for twelve samples collected in one aquaculture facility (Figure 2). The reference material collected in the Ria de Aveiro lagoon was confirmed to be *C. gigas*. Moreover, the EU007507, EU007510 and EU007512 sequences [19] (which were previously deposited in GenBank as *C. gigas*) nested as *C. angulata* in the phylogenetic analysis. Therefore, they were considered to be *C. angulata* in the subsequent analyses.

Such separation was confirmed by the median joining network (Figure S2). This network clearly evidenced the separation between *C. angulata* and *C. gigas*, as well as a clear-cut divergence of these two species from the congeneric *C. dianbaiensis*, *C. sikamea*, *C. nippona*, *C. virginica*, *C. ariakensis*, and (obviously) the two outgroups of *S. glomerata* and *S. cucullata*.

### 3.2. Haplotype Analysis and Genetic Diversity of Portuguese Oyster Populations

**Figure 3:**
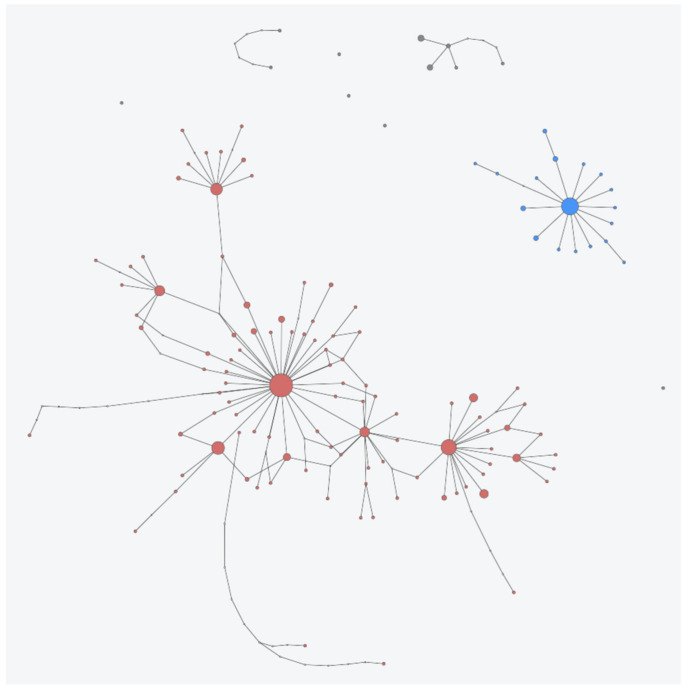
Haplotype minimum spanning network. Each circle represents a unique haplotype, each color represents each species as reported above (red, *C. angulata*; blue, *C. gigas*; grey, *C. dianbaiensis*, *C. sikamea*, *C. nippona*, *C. virginica*, *C. ariakensis*, and the two outgroups *S. glomerata* and *S. cucullata*), and the size of the circle is proportional to number of samples represented by each haplotype.

In total, 134 haplotypes were identified (Figure 3; Table S4): 104 for *C. angulata*; 18 for *C. gigas*; 5 for *C. dianbaiensis*; and one each for *C. sikamea*, *C. nippona*, *C. virginica*, *C. ariakensis*, *S. glomerata*, and *S. cucullata*. These haplotypes segregated into nine haplogroups: one for *C. angulata*; one for *C. gigas*; one each for *C. dianbaiensis*, *C. sikamea*, *C. nippona*, *C. virginica*, and *C. ariakensis*; and one each for outgroups *S. glomerata* and *S. cucullata*.

The obtained haplotype network confirmed the separation of the *C. angulata* and *C. gigas* haplogroups (Figure 3 and Table S4). Original samples of *C. gigas* collected in this study were included in five haplotypes, labelled as *Cr_gi01–Cr_gi05*, and deposited in GenBank under accession numbers OK021655–OK021659.

For *C. angulata*, 17 of the 104 described haplotypes included the original sequences collected from this study in the Sado estuary: haplotype sequences were labelled as *Cr_an01–Cr_an17* and deposited in GenBank under accession numbers OK030211–OK030227. Among them, five were newly identified (*Cr_an10*, *14*, *15*, *16*, and *17*) since they were never detected before among all *C. angulata* sequences available in GenBank.

Considering the frequency of the 104 haplotypes obtained for the Portuguese oyster, four haplotypes were the most common ones, including sequences collected both from the Indo-Pacific and from Portuguese and Spanish populations. In detail, haplotype *Cr_an01* (reference haplotype sequence: OK030211) comprised 75 sequences (biggest haplotype probability: 0.076), haplotype *Cr_an03* (reference haplotype sequence: OK030213) comprised 32 sequences (haplotype probability 0.038), haplotype *Cr_an08* (reference haplotype sequence: OK030218) comprised 22 sequences (haplotype probability: 0.047), and haplotype *Cr_an07* (reference haplotype sequence: OK030217) comprised 18 sequences (haplotype probability: 0.012) (Table S4).

However, most of the identified haplotypes (89 out of 104) were characterized by a single sequence (singletons) or two sequences; mainly, 73 were distributed in the original area of species distribution (China, Taiwan, Korea, and Japan), but 16 were identified in Portuguese populations.

**Figure 4:**
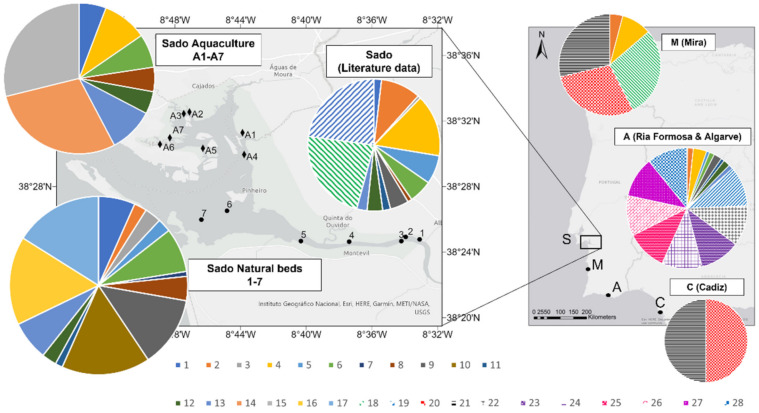
Distribution of the 28 haplotypes of *C. angulata* occurring in European populations. The original data of this study refer to the Sado aquaculture (A1–A7) and Sado natural oyster beds (1–7); all other data were collected from GenBank sequences. Haplotype numbers 1–17 (originally named *Cr_an01–Cr_an17)* refer to sequences OK030211–OK030227, haplotype 18 corresponds to reference sequence AY397686, haplotype 19 corresponds to reference sequence KY363483, haplotype 20 corresponds to reference sequence AJ553907, haplotype 21 corresponds to reference sequence AJ553908, and haplotypes 22–28 correspond to reference sequences MG209523-29.

As for their geographic distribution, among the global 104 haplotypes identified for Portuguese oysters, 28 were identified in the European populations in Portugal and Spain (Cadiz) (Figure 4).

Moreover, 19 of these haplotypes—21 if considering two additional haplotypes previously identified also in Spain (Cadiz)—only occurred in Portugal, namely in the Sado and Mira estuaries, Ria Formosa, and Algarve.

Eight haplotypes were shared among Indo-Pacific and Portuguese populations, whilst 75 haplotypes were never detected in Portuguese or Spanish populations (both original samples and previously deposited data).

Focusing on the original samples collected in this study from the Sado Estuary, the results obtained revealed some differences between the *C. angulata* samples belonging to natural oyster beds and aquaculture facilities (see Figure 4). The samples from the natural beds were characterized by the presence of 14 haplotypes: *Cr_an01–Cr_an03*, *Cr_an05– Cr_an13*, *Cr_an16*, and *Cr_an17*. Eight haplotypes were identified in farmed samples: *Cr_an01*, *Cr_an04*, *Cr_an06*, *Cr_an08*, *Cr_an12*, *Cr_an13*, *Cr_an14*, and *Cr_an15*. Five haplotypes were shared by both groups: *Cr_an01*, *Cr_an06*, *Cr_an08*, *Cr_an12*, and *Cr_an13*.

It is noteworthy that most of the described haplotypes were found to have a very low frequency, being represented only by one or a few sequences. A total of 45% of the sequences collected in the studied natural oyster beds and aquaculture facilities were grouped into haplotype *Cr_an01*, which was the most common one even when considering the entire *C. angulata* dataset (Figure 3 and Table S4). This could be useful to understand the Tajima Neutrality test, which showed a negative D parameter for all tested combinations: when applied to all *C. angulata* sequences (D = −2.23, ** *p* < 0.01); when only applied to the sequences obtained from Portuguese populations that also included GenBank data (D = −1.87, ** *p* < 0.05); and when limited to the sequences collected for the present study, although it was not significant in this case (D = −1.32, *p* > 0.10).

## 4. Discussion

This study allowed for the collection of *cox1* sequences of *C. angulata* from the Sado estuary from both natural oyster beds and aquaculture facilities, and their comparison with a wide dataset of cupped oyster sequences obtained from GenBank—to our knowledge the widest available for Portuguese oysters.

Our analysis confirmed that most of the original samples of this study could be taxonomically identified as *C. angulata*, except for a limited number of samples collected from an aquaculture facility that were identified as *C. gigas*. Moreover, results obtained from *cox1* sequence analyses confirmed the existence of a genetic distance between the two species, although it was lower than those occurring with all other species of the same genus.

The oyster populations of the Sado estuary showed high haplotypic variability and were characterized by five original haplotypes that were not previously described; however, most of them were found to have a very low frequency, being represented by only one or two sequences. Natural populations showed higher haplotypic variability compared to farmed ones. The sharing of five common haplotypes in both natural and cultivated oysters, two of which (*Cr_an01* and *Cr_an08*) showed higher frequencies, is compatible with the fishery activities that are carried out in the Sado estuary. A richness in singletons, i.e., pronounced/strong sweeps related to an excess of low frequency polymorphisms [40,41], and the high frequencies of very common haplotypes result in significantly negative values of the D Tajima’s statistics [38]. A negative D value can indicate a possible recent population expansion in the Sado estuary that is compatible with a founder effect related to the non-native origin of *C. angulata* or with a genetic drift and a bottleneck caused by the strong demographic reduction in the 1960s and 1970s and the recent recovery that shaped their current genetic structure and diversity. As previously underlined [42], this test is frequently used by conservation biologists due to its advantages, including the fact that the Tajima test can be performed on sequences belonging to any coding or noncoding locus of any species and no outgroup is required [42].

The haplotypic variability of the samples collected in natural oyster beds was an example of in situ sustainable management that clearly demonstrated the importance of the integrated conservation of wild populations. Their natural genetic diversity will constitute a fundamental source of variation and greater adaptation to the naturally variable conditions of the Sado estuary, reinforcing the viewpoint that aquaculture activities must ensure the high genetic diversity and fitness of cultivated stocks in a global change scenario.

Moreover, the absence of *C. gigas* from the sampled natural oyster beds is good news from the viewpoint of Portuguese oyster conservation, especially considering that *C. angulata* beds in the Sado estuary are among the last existing populations in Europe and are considered the purest ones in terms of introgression with the Pacific oyster [2]. The conservation of the ancestral genetic traits of *C. angulata* may be due to the fact that the introduction of cultured *C. gigas* in the estuary has been forbidden by local authorities, a situation that has not taken place in the Ria Formosa and the Ria de Aveiro. In fact, recent genomic data [2] showed that the Ria Formosa population has higher level of introgression with genetic traits of *C. gigas* because of Pacific oyster cultivation that has been conducted for more than 15 years [2,43,44].

Nevertheless, the data obtained in this study showed that specimens of *C. gigas* are cultivated in the local aquaculture facilities. These results were corroborated by interviews with local oyster producers, who confirmed that some of them import oyster seeds from France [45]. The cultivation of the Pacific oyster represents a concrete risk to Portuguese oyster conservation due to its ability to hybridize. In fact, though the genetic divergence between these two species is low, they show phenotypic differences, including in their resistance to diseases, growth rates, and physiological behavior [2], that are particularly relevant in terms of biodiversity conservation and aquaculture production. The introduction of the Pacific oyster could also represent a risk for the spreading of new pathogens. Unfortunately, this last risk became real in several Portuguese systems where the Pacific oyster was introduced. The severe outbreaks of a *Herpes* virus that have occurred in France since 2012 reached the Portuguese populations in 2014, 2015, and 2016 with mortality rates close to 90%. Therefore, since 2008, the Portuguese authorities have forbidden the introduction of the Pacific oyster into the Natural Reserve of the Sado Estuary to preserve the Portuguese oyster beds. However, as highlighted by the results of this paper, the Pacific oyster occurred in at least one of the investigated aquaculture facilities.

It is therefore clear that the conservation of last “pure” populations of *C. angulata* from the Sado estuary should be considered a priority, especially for local authorities and oyster producers, due to their biological, ecological, cultural, and economic value. Efforts should be made to tightly regulate the introduction of *C. gigas* in both natural beds and aquaculture facilities in order to prevent the hybridization of the valuable Portuguese oysters with the invasive Pacific ones.

It is noteworthy that interviews conducted with the oyster producers in the Sado estuary have indicated that the “certified origin” of the product and the creation of a “Sado label” are two of the most important measures to improve oyster production and cultivation [45].

Additionally, the specific regulations regarding the use of NIS in aquaculture [46] and the specific restrictions and measures required for NIS of EU concern [47] should be implemented to effectively mitigate the risk [14]. Therefore, the monitoring and restoration of Portuguese oyster populations, with reference to the Sado estuary, should be regularly carried out, as previously suggested [2].

## 5. Conclusions

Although the Portuguese oyster cannot be strictly described as a native species of Portugal, it was introduced a long time ago and is an important component of the estuarine habitats, with a relevant cultural and economic value. Therefore, the conservation of the last reference populations of *C. angulata* should be considered a priority, both in the Natural Reserve of the Sado estuary and in the Mira estuary, another estuarine system included in a protected area.

Genetic data obtained from this study confirmed that the Pacific oyster occasionally occurs in oyster aquaculture facilities but does not occur in the natural oyster beds of the Sado estuary. However, the presence of the hybridizing congeneric represents a concrete problem, and risks are arising for the conservation of the Portuguese oyster reference populations of the Sado estuary: therefore, researchers, local authorities, and oyster producers should work together to avoid the loss of this valuable resource.

## Data Availability

Original data from this study have been deposited in GenBank under Accession Numbers OK021655–OK021659 and OK030211–OK030227.

## Supplementary Materials

The following are available online at https://www.mdpi.com/article/10.3390/life11111173/s1, Figure S1: substitution saturation test: a) first codon position; b) second codon position; c) third codon position. Figure S2: Median-joining network constructed using PopArt 1.7 based on 394 sequences. Each circle represents a unique genome/group of affine genomes, the colors represent the species, as reported above (red, *C. angulata*; blue, *C. gigas*; grey, *C. dianbaiensis*, *C. sikamea*, *C. nippona*, *C. virginica*, *C. ariakensis*, and the two outgroups *S. glomerata* and *S. cucullate*), and the size of the circle is proportional to number of genomes included in each circle. Figure legend represents dimension proportionality to sample number included in each circle and colour correspondence. Numbers of temples (one line per mutation) represent the number of single nucleotide variations (SNVs) between groups. Table S1: GenBank sequences included in the alignment and phylogenetic analyses. For each sequence the species, the GenBank accession number, the original source, and the sampling location (if available) are provided. The sequences marked with (*) A.N. EU007507, 510, and 512 were deposited in GenBank as *C. gigas*, but the phylogenetic analyses conducted here confirmed that they belong to *C. angulata*. Previous data collected from Sado Estuary are marked in bold. Table S2: List of original samples sequenced in this study. Table S3: Test of substitution saturation (Xia and Lemey, 2009, Xia et al., 2003). If Iss was significantly smaller than Iss.c, only a little saturation was observed. Iss.cSym was Iss.c when assuming a symmetrical topology; Iss.cAsym was Iss.c when assuming an asymmetrical topology. Given the large number of OTUs in the present dataset, only the results for the highest number of OTUs (32) are shown; P was estimated to be 0.0000 in all cases. Table S4: Haplotype analysis of all the sequences included in the final alignment. Haplogroup/species; haplotype name with reference sequence; specimens/sequences for each haplotype are indicated. Original haplotypes from this study are indicated in bold.
