# Supplementary material for: One in a Million: Genetic Diversity and Conservation of the Reference Crassostrea angulata Population in Europe from the Sado Estuary (Portugal)
Source: Life (Basel). 2021 Nov 3;11(11):1173. doi: 10.3390/life11111173 (PMC8625788; doi:10.3390/life11111173)
Supplement: Supplementary file 1 [file life-11-01173-s001.zip › life-1391426-supplementary/life-1391426-supplementary-for conversion/Chiesa et al., Supplementary Table S1.pdf]

life

MDPI

Table S1. GenBank sequences included in the alignment and phylogenetic analyses. For each sequence the species, the GenBank accession number, the original source and the sampling location (if available) are provided. The sequences marked with (*) A.N. EU007507, 510 and 512 were deposited in GenBank as *C. gigas*, but the phylogenetic analyses herein conducted confirmed that they belong to *C. angulata.*

Species	Accession Number	Source	Sampling Site (if Available)
Crassostrea angulata	DQ659372-74	Direct submission (Cardoso et al., 2006)	**Sado estuary (Portugal)**
	AB904879-83; 85-88; 90	Direct submission (Hamaguchi et al., 2014)	Amani Island, Kagoshima (Japan)
	JQ027306-08	Direct submission (Hsiao, 2011)	Taiwan
	HQ661008-09	*Liu et al., 2011 Mol Ecol Res 11 (5): 820-830*	Yangjiang - Guandong; Pingtan -Fujjan (China)
	AF152567	*O' Foighil et al., 1998 Mar Biol 131: 497-503*	**Sado estuary (Portugal)**
	AY455664	*Lapègue et al., 2004 J Shell Res 23 (3): 759-763*	**Sado and Mira estuaries (Portugal)**
	AY397685-86	*Lapègue et al., 2004 J Shell Res 23 (3): 759-763*	**Sado and Mira estuaries (Portugal)**
	EU815987-96	Direct submission (Xia,J. and Yu,Z., 2008)	Southern Chinese Sea
	KY363472-83	*Batista et al., 2017 Mar Biol 164:110*	**Sado estuary and Ria Formosa (Portugal)**
	KT932101-09	*Moreira et al., 2016 Stoten 545–546: 569–581*	**Sado estuary (Portugal)**
	KU726888-6939	*Hsiao et al., 2016 Scientific Reports 6:34057*	Taiwanese coast and Chinese coast
	KP216768-6806	Direct submission (Li et et al., 2016)	China
	MH938693	Direct submission (Wu and Zang, 2019)	Sanmen Bay, Wenzhou, Zhejiang Province (China)
	MG209523-29	Direct submission (Garcia-Vazquez,E. and Borrell Pichs, 2018)	Faro, Algarve (Portugal)
	KX345754-59	Direct submission (Hu,L. and Wang, 2017)	China
	KU947169-186	Direct submission (Li,H. and Xu,Z, 2016)	Daya Bay (China)
	KP067886-89	Direct submission (Park,J.K. and Lee, 2015)	Korea
	AJ553907-08	*Boudry et a al., 2003*	Mira estuary (Portugal) and Cadiz (Spain)

		Aquaculture 228 (2003) 15–25	
	LC123936-50	Direct submission (Sekino et al., 2016)	Shikoku Island (Japan)
	LC383458-67	Direct submission (Hamaguchi et al., 2018)	southern part of Wakayama Prefecture (Japan)
	EU672832	Direct submission (Ren et al., 2016)	China: Taiwan
	FJ841965	*Wu et al., 2010 Mol Phylogenet Evol 57 (1), 448-454*	Dianbai, Guangdong Province (China)
	NC_012648	Direct submission (Ren et al., 2009)	China: Taiwan
	KJ855246-47; KJ855249	*Ren et al., 2016 Mar. Biotechnol. 18 (2), 242-254*	(China)
	KJ855248	*Ren et al., 2016 Mar. Biotechnol. 18 (2), 242-254*	Portugal
	KC683507	Direct submission (Li and Wang, 2018)	Guangxi, China
	KX579088-91	Direct submission (Luo and Zhang, 2017)	Southeast coast of China
	KC683500-01	Direct submission (Li and Wang, 2014)	Coastal sea in Guangxi (China)
	AB736846-47	*Sekino et al., 2012; Mar. Biol. 159, 2757-2776*	Shangdon, Qingdao (China)
	EU815987	Direct submission (Xia and Yu, 2016)	South China Sea
	KC170323	Direct submission (Wang, 2013)	China: Houhai Bay, Putian, Fujian
Crassostrea dianbaiensis	LC123923-35	Direct submission (Sekino et al., 2016)	Shikoku Island (Japan)
Crassostrea gigas	AB904889	Direct submission (Hamaguchi et al., 2014)	Amani Island, Kagoshima, (Japan)
	HQ661002-03	*Liu et al., 2011 Mol Ecol Res 11 (5): 820-830*	Rongcheng, Shandong (China)
	HQ661004	*Liu et al., 2011 Mol Ecol Res 11 (5): 820-830*	Qingdao, Shandong (China)
	HQ661005	*Liu et al., 2011 Mol Ecol Res 11 (5): 820-830*	Yantai, Shandong (China)
	HQ661006-7	*Liu et al., 2011 Mol Ecol Res 11 (5): 820-830*	Lianyungang, Jiangsu (China)
	AF152565	*O' Foighil et al., 1998 Mar Biol 131: 497-503*	Hokkaido, Kyshu, Hiroshima (Japan)

	DQ417690-91	*Cardoso et al., 2007 J. Sea Res. 57, 303-31*	Wadden Sea (Holland)
	DQ417692-93	*Cardoso et al., 2007 J. Sea Res. 57, 303-31*	Oosterschelde estuary (Holland)
	DQ417694; 96	*Cardoso et al., 2007 J. Sea Res. 57, 303-31*	La Rochelle (France)
	DQ659371	Direct submission (Cardoso et al., 2006)	Wadden Sea (Holland)
	**EU007507* (phylogenetically identified as *C. angulata*)**	*Reece et al., 2008 Mar. Biol. 153 (4), 709-721*	China
	EU007508	*Reece et al., 2008 Mar. Biol. 153 (4), 709-721*	Japan
	EU007509; 11	*Reece et al., 2008 Mar. Biol. 153 (4), 709-721*	USA
	**EU007510* (phylogenetically identified as *C. angulata)***	*Reece et al., 2008 Mar. Biol. 153 (4), 709-721*	China
	**EU007512* (phylogenetically identified as *C. angulata*)**	*Reece et al., 2008 Mar. Biol. 153 (4), 709-721*	China
	JF700177	Direct submission (Zhang et al., 2011)	Not specified
	KF643519	*Layton et al., 2014 PLosOne 9 (4), E95003*	British Columbia, Nanaimo (Canada)
	KF643604	*Layton et al., 2014 PLosOne 9 (4), E95003*	British Columbia, Nanaimo (Canada)
	KF643857	*Layton et al., 2014 PLosOne 9 (4), E95003*	British Columbia, Nanaimo (Canada)
	KF644048	*Layton et al., 2014 PLosOne 9 (4), E95003*	British Columbia, Nanaimo (Canada)
	KY363466-71	*Batista et al., 2017 Mar Biol 164:110*	Seudre River (France)
	KT932093-2100	*Moreira et al., 2016 Stoten 545–546: 569–581*	Aveiro Lagoon (Portugal)
	AJ553909-11	*Boudry et a al., 2003 Aquaculture 228 (2003) 15–25*	Bangor (United Kingdom), Gravelines (France)
Crassostea virginica	EU007485	*Reece et al., 2008 Mar. Biol. 153 (4), 709-721*	USA
	AF152566	*O′ Foighil et al., 1998 Mar Biol 131: 497-503*	Delaware Bay (USA)
Crassotrea sikamea	AF152568	*O′ Foighil et al., 1998 Mar Biol 131: 497-503*	Tomales Bay (California) and Yaquina Bay (Oregon) (USA)
Crassostea ariakensis	AF152569	*O′ Foighil et al., 1998 Mar Biol 131: 497-503*	Washington State (USA)
Crassostea nippona	AF300616	*Lee et al., 2000 Korean J. Biol. Sci. 16 (2), 203-211*	Not specified

Saccostrea glomerata	EU007482	*Reece et al., 2008 Mar. Biol. 153 (4), 709-721*	Australia
Saccostrea cucullata	AY038076	*Klinbunga et al. (2003) Mar Biol Biotech 5: 27-36*	Thailand

Reference

1. Liu, J.; Li, Q.; Kong, L.; Yu, H.; Zheng, X. Identifying the true oysters (Bivalvia: Ostreidae) with mitochondrial phylogeny and distance-based DNA barcoding *Mol. Ecol. Resour*. **2011**, *11*, 820–830.
2. O′ Foighil, D.; Gaffney, P.M.; Wilbur, A.E.; Hilbish, T.J. Mitochondrial cytochrome oxidase I gene sequences support an Asian origin for the Portuguese oyster *Crassostrea angulata*. *Mar Biol*. **1998**, *131*, 497–503.
3. Lapègue, S.; Batista, F.M.; Heurtebise, S.; Yu, Z.; Boudry, P. Evidence for the presence of the Portuguese oyster, *Crassostrea angulata,* in northern China. *J. Shellfish. Res.* **2004**, *23*, 759–763.
4. Batista, F.M.; Fonseca, V.G.; Ruano, F.; Boudry, P. Asynchrony in settlement time between the closely related oysters *Crassostrea angulata* and *C. gigas* in Ria Formosa lagoon (Portugal). *Mar. Biol.* **2017**, *164*, 110.
5. Moreira, A.; Figueira, E.; Soares, A.M.; Freitas, R. The effects of arsenic and seawater acidification on antioxidant and biomineralization responses in two closely related *Crassostrea* species *Sci. Total Environ.* **2016**, *545–546*, 569–581.
6. Hsiao, S.T.; Chuang, S.C.; Chen, K.S.; Ho, P.H.; Wu, C.L.; Chen, C.A. DNA barcoding reveals that the common cupped oyster in Taiwan is the Portuguese oyster *Crassostrea angulata* (Ostreoida; Ostreidae), not *C. gigas*. *Sci. Rep*. **2016**, *6*, 34057.
7. Boudry, P.; Heurtebise, S.; Lapègue, S. Mitochondrial and nuclear DNA sequence variation of presumed *Crassostrea gigas* and *C. angulata* specimens: A new oyster species in Hong Kong? *Aquaculture* **2003**, *228*, 15–25.
8. Wu, X.; Xu, X.; Yu, Z.; Wei, Z.; Xia, J. Comparison of seven *Crassostrea* mitogenomes and phylogenetic analyses. *Mol. Phylogenet. Evol.* **2010**, *57*, 448–454.
9. Ren, J.; Hou, Z.; Wang, H.; Sun, M.A.; Liu, X.; Liu, B.; Guo, X. Intraspecific Variation in Mitogenomes of Five *Crassostrea* Species Provides Insight into Oyster Diversification and Speciation. *Mar. Biotechnol.* **2016**, *18*, 242–254.
10. Sekino, M.; Sato, S.; Hong, J.S.; Li, Q. Contrasting pattern of mitochondrial population diversity between an estuarine bivalve, the Kumamoto oyster *Crassostrea sikamea*, and the closely related Pacific oyster *C. gigas Mar. Biol.* **2012**, *159*, 2757–2776.
11. Cardoso, J.F.; Langlet, D.; Loff, J.; Martins, A.; Witte, J.; Santos, P.T.; van der Veer, H.W. Spatial variability in growth and reproduction of the Pacific oyster *Crassostrea gigas* (Thunberg, 1793) along the west European coast. *J. Sea Res.* **2007**, *57*, 303–315.
12. Reece, K.S.; Cordes, J.F.; Stubbs, J.B.; Hudson, K.L.; Francis, E.A. Molecular phylogenies help resolve taxonomic confusion with Asian *Crassostrea* oyster species. *Mar. Biol*. **2008**, *153*, 709–721. doi:10.1007/s00227-007-0846-2.
13. Layton, K.K.; Martel, A.L.; Hebert, P.D. Patterns of DNA barcode variation in Canadian marine molluscs. *PLoS ONE* **2014**, *9*, E95003.
14. Lee, S.Y.; Park, D.W.; An, H.S.; Kim, S.H. Phylogenetic relationship among four species of Korean oysters based on mitochondrial *16S* rDNA and *CO1* gene. *Korean J. Biol. Sci.* **2000**, *16*, 203–211.
15. Klinbunga, S.; Khamnamtong, N.; Tassanakajon, A.; Puanglarp, N.; Jarayabhand, P.; Yoosukh. W. Molecular genetic identification tools for three commercially cultured oysters (*Crassostrea belcheri, Crassostrea iredalei*, and *Saccostrea cucullata*) in Thailand. *Mar. Biotech*. **2003**, *5*, 27–36.
