# Supplementary material for: One in a Million: Genetic Diversity and Conservation of the Reference Crassostrea angulata Population in Europe from the Sado Estuary (Portugal)
Source: Life (Basel). 2021 Nov 3;11(11):1173. doi: 10.3390/life11111173 (PMC8625788; doi:10.3390/life11111173)
Supplement: Supplementary file 1 [file life-11-01173-s001.zip › life-1391426-supplementary/life-1391426-supplementary-for conversion/Chiesa et al., Supplementary Table S3.pdf]

life

Table S3. Test of substitution saturation. Test of substitution saturation (Xia and Lemey, 2009, Xia et al., 2003). If Iss is significantly smaller than Iss.c, only little saturation is observed. Iss.cSym is Iss.c assuming a symmetrical topology; Iss.cAsym is Iss.c assuming an asymmetrical topology. Given the large number of OTUs in the present dataset, only result for the highest number of OTUs (32) is shown; P was estimated to be 0.0000 in all cases.

Codon Position		Iss	Iss.cSym	Iss.cAsym
1	0.050	0.698	0.391	***
2	0.045	0.698	0.691	***
3	0.148	0.698	0.391	***

Reference

1. Xia, X., Lemey, P. Assessing substitution saturation with DAMBE. In *The Phylogenetic Handbook: A Practical Approach to DNA and Protein Phylogeny*, 2nd ed.; Lemey, P., Salemi, M., Vandamme, A.M., Eds.; Cambridge University Press: Cambridge UK, 2009, pp. 615–630.
2. Xia, X.; Xie, Z.; Salemi, M.; Chen, L.; Wang, Y. An index of substitution saturation and its application. *Mol. Phylogenet. Evol.* **2003**, *26*, 1–7, doi:10.1016/s1055-7903(02)00326-3.
