# Supplementary figures and images for: One in a Million: Genetic Diversity and Conservation of the Reference Crassostrea angulata Population in Europe from the Sado Estuary (Portugal)
Source: Life (Basel). 2021 Nov 3;11(11):1173. doi: 10.3390/life11111173 (PMC8625788; doi:10.3390/life11111173)

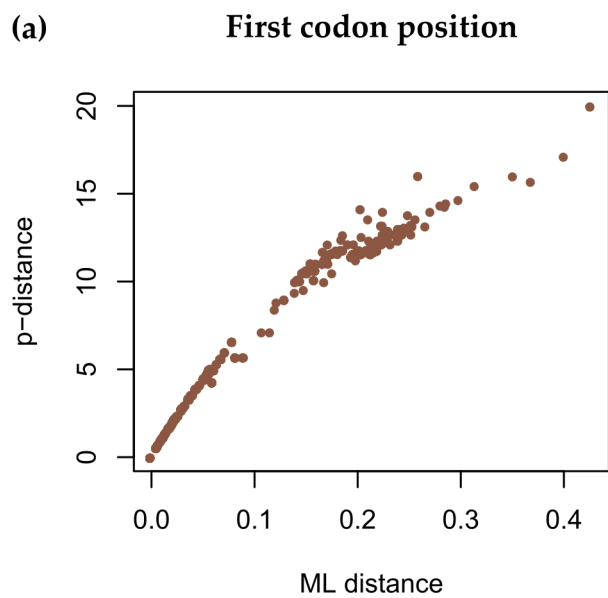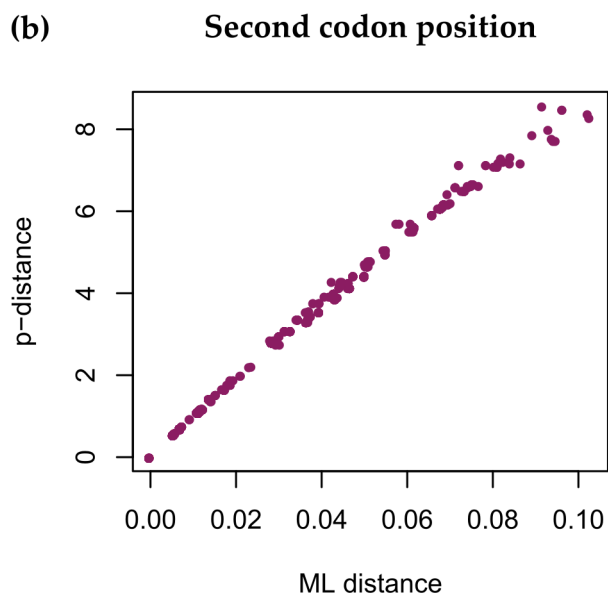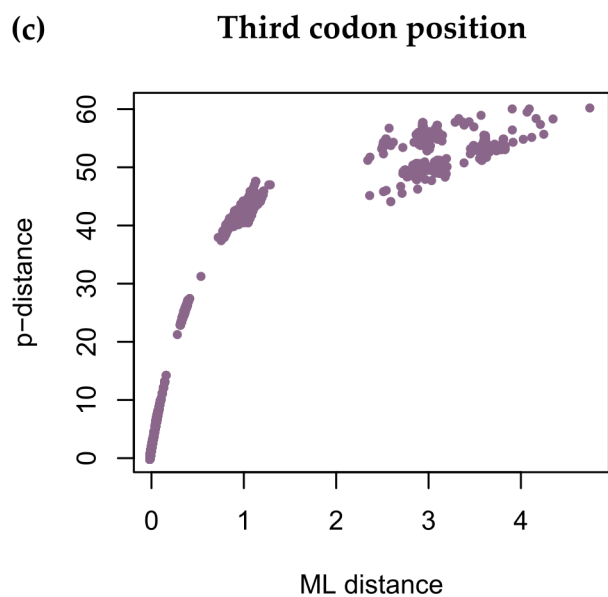

Supplement: Supplementary file 1 [file life-11-01173-s001.zip › life-1391426-supplementary/life-1391426-supplementary-for conversion/Chiesa et al., Supplementary Fig S1.pdf]

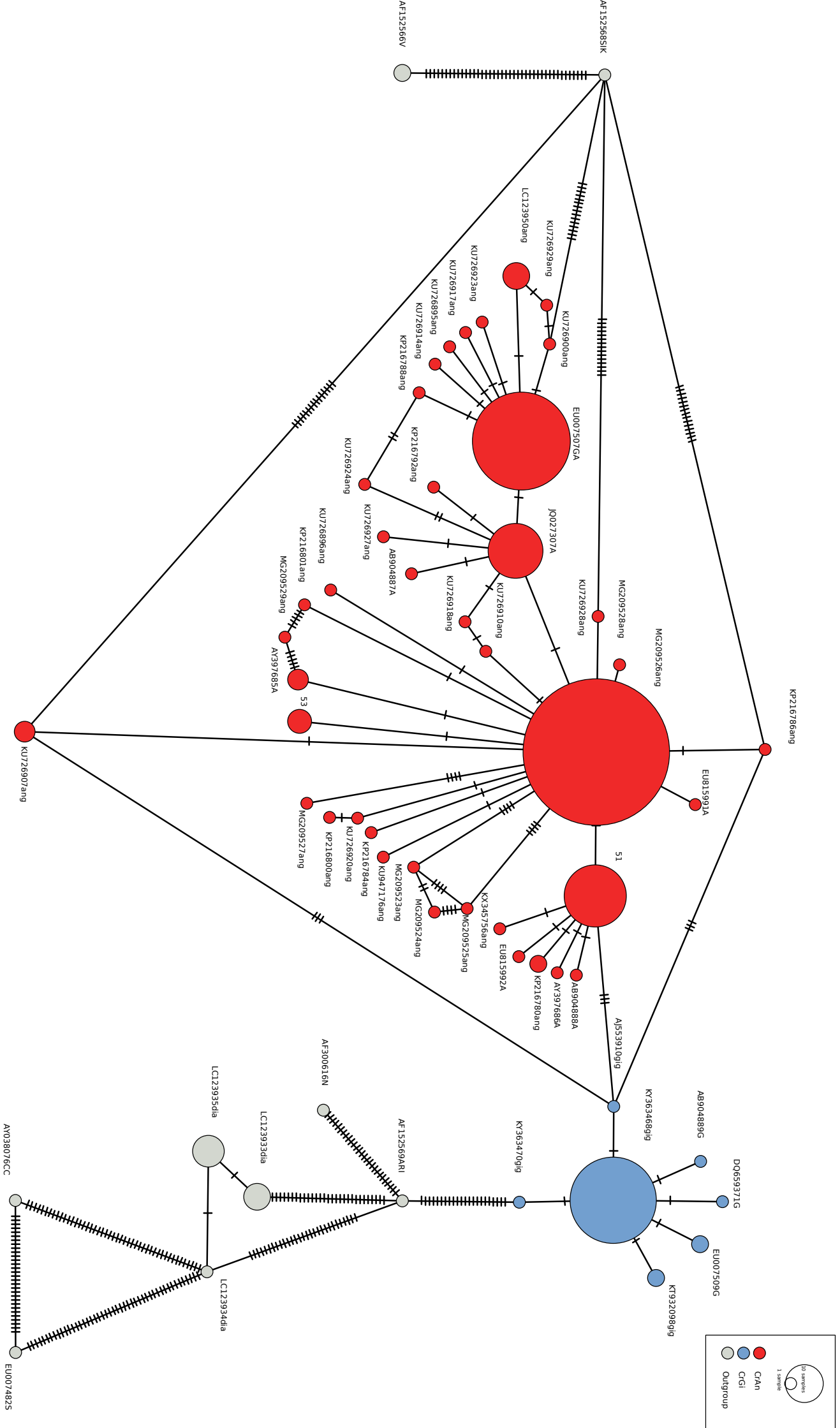

Supplement: Supplementary file 1 [file life-11-01173-s001.zip › life-1391426-supplementary/life-1391426-supplementary-for conversion/Chiesa et al., Supplementary Fig S2.pdf]
